# A Wilms' Tumor with Spinal Cord Compression: An Extrarenal Origin?

**DOI:** 10.1155/2018/1709271

**Published:** 2018-09-03

**Authors:** Audrey Petit, Amandine Rubio, Chantal Durand, Christian Piolat, Cécile Perret, Anne Pagnier, Dominique Plantaz, Hervé Sartelet

**Affiliations:** ^1^Département de Pédiatrique, CHU de Grenoble, Grenoble, France; ^2^Département de Radiologie, CHU de Grenoble, Grenoble, France; ^3^Département de Chirurgie, CHU de Grenoble, Grenoble, France; ^4^Département de Pathologie, CHU de Grenoble, Grenoble, France

## Abstract

Spinal cord compression in Wilms' tumor (WT) is an extremely rare event that can have a very poor prognosis if not taken care of rapidly. Most cases reported in the literature involve widely metastatic patient with bone or paraspinal metastases or occasionally intradural metastasis. Here, we present the case of a 3-year-old girl of WT confirmed by biopsy, with spinal cord compression due to the direct contiguous spread of a tumor through 2 vertebral foramina. Abdominal ultrasonography and magnetic resonance imaging performed for an abdominal mass revealed a large heterogeneous tumor near the upper pole of the left kidney. A nodular infiltration extended through the T11-L1 and L1-L2 neural foramina, forming an intraspinal mass that compressed the spinal cord. Major paresthesia subsequently occurred, requiring urgent treatment with corticosteroids and chemotherapy. The evolution was rapidly satisfying. After six courses of chemotherapy, a left nephrectomy was performed. Macroscopic examination identified a large tumor attached to the kidney without renal infiltration. Microscopical examination concluded to a nephroblastoma with regressive changes, of intermediate risk. Evolution at 6 months is satisfactory, with no neurological deficit. The histological aspect of the tumor and the clinical outcome suggest that she had an extrarenal WT that spread through the vertebral foramina and was secondarily attached to the kidney.

## 1. Introduction

Childhood renal neoplasm accounts for approximately 7% of all cancers in childhood and are in the vast majority Wilms' tumor (WT) or nephroblastoma [1, 2]. About 10% of WT present with heamatogenous spread, most commonly to the lungs (85%), liver (10%) and only very rarely to the bones (1%) and brain [1, 2]. The occurrence of spinal cord compression ranges from 2.7 to 4% in childhood neoplasm, generally in metastatic or invasive Ewing's sarcoma, osteogenic sarcoma, rhabdomyosarcoma, neuroblastoma, and lymphoma [3, 4]. Spinal cord compression may result in permanent neurological deficit, further aggravating the burden of disease.

In the course of WT, spinal cord compression is a very rare occurrence, usually involving skeletal metastases to the vertebral body, intradural or extradural metastases [5–11].

Here, we report the case of a large WT in a 3-year-old patient with secondary spinal compression by direct contiguous spread through 2 vertebral foramina.

## 2. Case Presentation

A 3-year-old girl, with no prior medical history, was admitted in our center with a three-week history of an abdominal mass discovered by her mother. On physical examination, a firm, painless mass in the left flank was palpable. Complete examination showed no other abnormality. In particular, no neurological deficit was detected.

**Figure 1:**
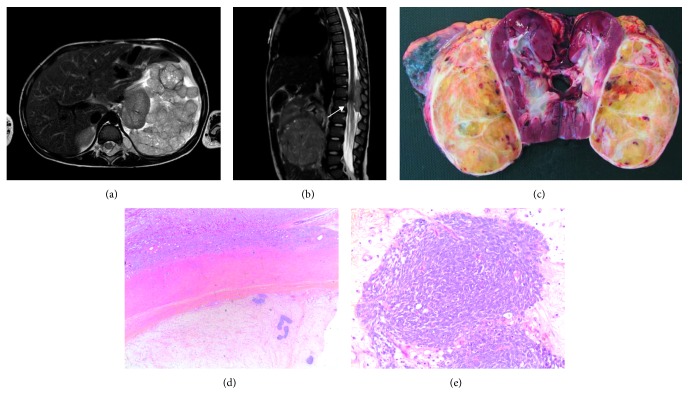
(a) Magnetic resonance imaging (MRI) showed an encapsulated tumor, with signs of capsular effraction at the upper pole of the left kidney. (b) Magnetic resonance imaging (MRI) showed a tumor, forming an intraspinal mass from T11 to L1 and compressing the spinal cord (arrow). (c) The left nephrectomy presented macroscopically a large tumor attached to the kidney without renal infiltration. (d) The microscopical examination confirmed the separation between the tumor and the kidney (Hematein-Eosin-Safran, ×100). (e) Wilm's tumor, with regressive changes, of intermediate risk with only small areas of blastema (Hematein-Eosin-Safran, ×400).

Abdominal ultrasonography revealed a large heterogeneous tumor of 69 × 67 ×  97 cm originating from the upper pole of the left kidney, deviating it towards the midline. The mass is located on the periphery of the upper pole of the kidney, and a vascular pedicle seemed to emerge from the renal sinus. No calcification or hemorrhagic component was found. Magnetic resonance imaging (MRI) and computed tomography (CT) showed an encapsulated tumor but with a nodular infiltration of the retroperitoneal fatty tissues. It extended through the T11-T12 and T12-L1 neural foramina, forming an intraspinal mass from T11 to L1 and compressing the spinal cord (Figure 1). Assessment of tumor extension revealed two infracentimetric metastases in the lungs. The tumor and its extradural extension showed a major hypermetabolic activity on positron emission tomography (PET). Bone marrow aspiration uncovered no medullary involvement. The urine catecholamines, neural specific enolase, alpha-foetoprotein, and human chorionic gonadotropin were normal. Laboratory studies evidenced only a small rise in LDH (417 IU/L) and fibrinogen (7.2 g/L).

Considering this extremely unusual clinicoradiological presentation, a posterior transcutaneous needle biopsy was performed, as recommended in the International Society of Pediatric Oncology renal study group SIOP-RTSG 2001 protocol. The histopathologic features revealed a triphasic nephroblastoma, with no anaplastic feature.

Meanwhile, the patient started complaining of major paresthesia and leg pain, requiring urgent treatment with corticosteroids and chemotherapy. Due to the neurological threat and the lung nodules, chemotherapy according to the SIOP-RTSG 2001 for stage IV nephroblastoma was administered, including three drugs (vincristine, actinomycine D, and doxorubicine).

The patient's evolution was rapidly satisfying, with the rapid and complete receding of neurological symptoms. The preoperative assessment, after four courses of chemotherapy, indicated a massive regression of the tumor volume by 53%, with measures of 67 × 46 × 77 cm, and a complete disappearance of the intraspinal extension. The lung nodules were no longer detected on CT imaging.

After six courses of chemotherapy, a left nephrectomy was performed. Macroscopic examination identified a large tumor attached to the kidney, enclosed in a thick fibrous capsule. The microscopic examination concluded to a triphasic nephroblastoma with regressive changes, of intermediate risk and without capsular rupture, thereby staging it as a stage I of the SIOP-RTSG 2001 classification. The tumor consisted in tumor epithelial component (abortive tubules and glomeruli) surrounded by metanephric blastema and tumor immature spindled cell stroma without any anaplasia or emboli of tumor cells. The histology of the kidney was unremarkable without any nephrogenic rest. Postoperative treatment included 29 weeks of chemotherapy with the same three drugs. After 24 months of evolution, the child is in good health and has no neurological deficit.

## 3. Discussion

The occurrence of spinal cord compression in childhood neoplasm ranges from 2.7 to 4% and is most often seen in the terminal phase of a widely metastatic cancer [5–8] Although rare cases of intraspinal and vertebrae metastasis have been reported in WT, intraspinal extension by direct contiguous spreading in a child devoid of spinal dysraphism has very infrequently been described. Here, we reported a case of “dumbbell” WT extending through 2 neural foramina and forming an intraspinal mass from T11 to L1 with spinal cord compression in a 3-year-old child.

The most common tumors causing spinal cord compression are neuroblastomas, soft-tissue sarcomas, followed by osteogenic and Ewing sarcomas, lymphomas, and very rarely leukemia and WT [3, 4]. The precise pathogenesis of the spinal cord compression is variable depending on the type of tumor. Neuroblastoma is particularly prone to develop a spinal cord compression via direct contiguous spread from paravertebral disease due to its sympathetic origin, with a classical dumbbell aspect as was evidenced here [12]. The most frequently reported symptoms of spinal compression include back pain, weakness, sensory loss with gait disturbance, sphincter, and autonomic dysfunction [3]. Early signs of cord compression must imperatively be recognized, as prompt diagnosis and treatment are mandatory to decrease the risk of irreversible loss of neurologic function [3, 4].

In the course of WT, spinal cord compression is invariably explained by skeletal metastasis to the vertebral body, intradural or extradural metastasis, although solitary metastases to the spine have been described [5–11]. Most cases involve patients with widely metastatic diseases with a very poor prognosis [8]. Surviving patients often retain functional neurological deficits [8, 11].

Only two cases of spinal cord compression by contiguous spread of a WT through the neural foramina had previously been described [13, 14]. Both cases also occurred in extrarenal WT (ERWT), which is a very rare form of WT, estimated to account for less than 1% of all cases of WT. The hypothesized pathogenesis of ERWT is the development of ectopic nephrogenic rests into a nephroblastoma. Its prognosis is rather good with mostly a favorable histology and an 11% of local recurrence rate [15]. Cojean et al. reported the case of a 2-month-old boy who developed an abdominal ERWT extending through the intervertebral foramina, encroaching the spinal cord [13]. The other case, described by Govender et al., involved a child with preexistent occult spinal dysraphism, which facilitated the extension of the ERWT to the spinal canal [14].

Our patient did not have any spinal dysraphism. We hypothesize that she had an ERWT that spread through the vertebral foramina and was secondarily attached to the kidney. Indeed the macroscopic description of this tumor, with no renal capsular effraction, fits the description of an ERWT, even though no nephrogenic rests were found. The best outcome of this patient could be further evidence of the extrarenal origin of this neoplasm.

Spinal cord compression is a very rare occurrence in WT, but it can have dramatic functional and vital consequences if not taken care of appropriately. Treatment comprises of immediate chemotherapy and corticosteroids to reduce the tumor size, followed by nephrectomy.

While the vast majority of WT cases with spinal cord compression are explained by metastasis to the spinal canal (bone, extradural or intradural metastasis), we report the first case of contiguous spreading from the primary tumor through the neural foramina in a child devoid of spinal dysraphism. This case could be explained by the extrarenal origin of the nephroblastoma.
